# Universal thermal climate index in the Arctic in an era of climate change: Alaska and Chukotka as a case study

**DOI:** 10.1007/s00484-023-02531-2

**Published:** 2023-08-12

**Authors:** E.A. Grigorieva, V.A. Alexeev, J.E. Walsh

**Affiliations:** 1 Humboldt Universitat zu Berlin (HU), Berlin, Germany; 2 International Arctic Research Center, University of Alaska Fairbanks (IARC UAF), Fairbanks, USA

**Keywords:** Universal thermal climate index, Climatology, Temporal dynamics, Alaska, Chukotka

## Abstract

The modern unambiguous climate change reveals in a rapid increase of air temperature, which is more distinctly expressed in the Arctic than in any other part of the world, affecting people health and well-being. The main objective of the current research is to explore the inter- and intra-annual changes in thermal stress for people in the Arctic, specifically for two parts of Beringia: Alaska, USA, and Chukotka, Russia, using climatology of the universal thermal climate index (UTCI). Data for 39 locations are taken from the ERA5-HEAT reanalysis for the period 1979–2020. Climatologically, the study area is divided into four subregions in Alaska: North, Interior, West and South, and two in Chukotka: Interior and Coast. The extreme coldest UTCI categories (1 and 2) are most common in coastal locations of northern Alaska and Chukotka, where strong winds exacerbate the low temperatures during winter. The results show that the frequency of category 1 (UTCI<−40°C) varies spatially from a quarter of all hours annually in Alaska North to almost zero in Alaska South. On the other hand, the warmest categories are rarely reached almost everywhere in Alaska and Chukotka, and even categories 7 and 8 (UTCI between +26 and +38°C) are found occasionally only at interior locations. Category 6 with no thermal stress (UTCI between +9 and+26°C) has frequencies up to 3% and 25% in Alaska North and Interior, respectively. The extremely cold thermal stress frequencies have substantially decreased over the 1979–2020 period, especially in Alaska North and Chukotka Coast. At the same time, the number of hours with UTCI in the comfortable category of thermal perception has increased depending on subregion, from 25 to 203 h/year. Overall, a decrease in the UTCI categories of extremely cold stress is coupled with an increase in the comfortable range in both Alaska and Chukotka. The salient conclusion is that, from the point of view of comfort and safety, global warming has a positive impact on the climatology of thermal stress in the Arctic, providing advantages for the development of tourism and recreation.

**Supplementary Information:**

The online version contains supplementary material available at 10.1007/s00484-023-02531-2.

## Introduction

Ongoing global climate change is unequivocal (IPCC 2021), manifesting itself in an increase of ambient temperature, with the strongest and the most serious evidence of warming in the high northern latitudes (Walsh 2021; Przybylak et al. 2022). In the Arctic, it has wide-ranging implications for indigenous and non-indigenous inhabitants, causing direct and indirect health effects (Casson et al. 2019; Contosta et al. 2019), which may decrease the incidence of hypothermia and associated morbidity and mortality among the Arctic residents in winter (Donatuto et al. 2020). Climate change is associated with changes in extreme weather events, including both heat waves and cold spells, which can seriously change subsistence hunting and fishing of indigenous populations, reducing access to traditional foods (Herman-Mercer et al. 2020).

The thermal load of the environment on the human body is an important indicator of climate discomfort, especially in the harsh environments of the Arctic where extremely cold situations are often combined with the strong wind. According to observations in the Russian Arctic in 1973–2012, the lowest temperatures were recorded in the Severnaya Zemlya, in the Laptev Sea and on the Novosibirsk Islands, up to −40° …−45°C, in some years up to −50°C. At the same time, wind speed here can be very strong, higher than 15 m s^−1^ (Alexeev 2014).

Climate warming can seriously change bioclimate, potentially decreasing the period with exceptionally low temperatures on one hand, but improving the intervals with comfortable conditions, which are very important for both inhabitants (Mölders 2019; Konstantinov et al. 2020) and tourists traveling to the Arctic (Huang et al. 2021). This change in bioclimate is very important, especially given the growing demand for tourism development in the Arctic, and in particular in Alaska and Chukotka. The tourism sector here is small, but is expected to grow rapidly in the future, namely cruise and aviation tourism, adventure tourism, ecotourism—observation of attractions such as glaciers, coastal cliffs, waterfalls, and (or) birds and marine mammals such as whales and polar bear (Drage et al. 2022; Schwoerer and Dawson 2022; Wienrich and Lukyanova 2022; Lau et al. 2023).

Human health and well-being are subject to many influences, including the thermal state of the physical environment. The thermal state depends on various atmospheric variables such as air temperature, wind, humidity, and solar radiation in addition to physiological and behavioral variables, which include activity levels, clothing, a person’s posture, and underlying physical condition. The multiplicity of factors and their interrelationships have led to the development of various unitary indices (de Freitas and Grigorieva 2017). The universal thermal climate index (UTCI) is a recent index increasingly used to address human comfort in the context of climate and weather (Bröde et al. 2012; Potchter et al. 2018; Bröde 2021). The UTCI has many advantages and shortcomings (Błażejczyk et al. 2012; Błażejczyk and Kuchcik 2021). Among other considerations, the UTCI has important advantages: (i) it is expressed in °C—a unit of measurement that is understandable not only to specialists, but also to the broader public; and (ii) it can be used in a huge temperature range from below −50°C and up to higher than +50°C (Błażejczyk et al. 2012; de Freitas and Grigorieva 2017; Błażejczyk and Kuchcik 2021) and applied around the world, enabling direct comparisons of results for different areas: in Europe (Antonescu et al. 2021), in the Caribbean (Di Napoli et al. 2022), in the Arctic (Mölders 2019; Shartova et al. 2019; Huang et al. 2021), China (Mi et al. 2020; An et al. 2021; Wang and Yi 2021; Liu et al. 2022), Japan (Ohashi et al. 2018), Russia (Shartova et al. 2019; Konstantinov et al. 2020; Vinogradova 2021). The UTCI has been used in a variety of applications (Krüger 2021), including medical science (Shartova et al. 2019; Urban et al. 2021; Romaszko et al. 2022), tourism (Huang et al. 2021), and in urban planning (Krüger 2021).

In recent studies, Mölders (2019) used observational data from a set of several hundred stations in northern North America and Russia to construct climatology of the UTCI, with a focus on Alaska; however, temporal variations and trends were not addressed. Vinogradova (2021) evaluated climatological and extreme values of the UTCI for a network of surface stations in Russia, but temporal variations and trends were not considered. Huang et al. (2021) documented Arctic-averaged variations of the UTCI computed from the ERA5 reanalysis, but study area was limited to 65–90°N, and most of results were presented as averages over all or parts of the land areas north of 65° N.

The present study documents the seasonal and interannual variability as well as secular trends of the UTCI in Arctic terrestrial subregions. The study area includes two regions in Beringia: Alaska, which is the north-western-most state in the USA, and Chukotsky Autonomous Okrug (Chukotka), which is the north-eastern-most region in Russia. Both regions have coasts on the Bering Sea, which was once the Bering land bridge between Chukotka and Alaska (Elias et al. 1996); both regions have interior areas in which a maritime or coastal influence in moderating climate is blocked by mountain ranges, which can be expressed in thermal stress and hence in UTCI. Because both regions have extreme climates characterized by very low winter temperatures and strong winds in some areas (Bieniek et al. 2012; Akimov et al. 2014; Alexeev 2014; Mölders 2019; Walsh 2021), we believe that the UTCI is a much more human-relevant climate metric than the actual air temperature in these areas.

## Materials and methods

### Study area

**Fig. 1:**
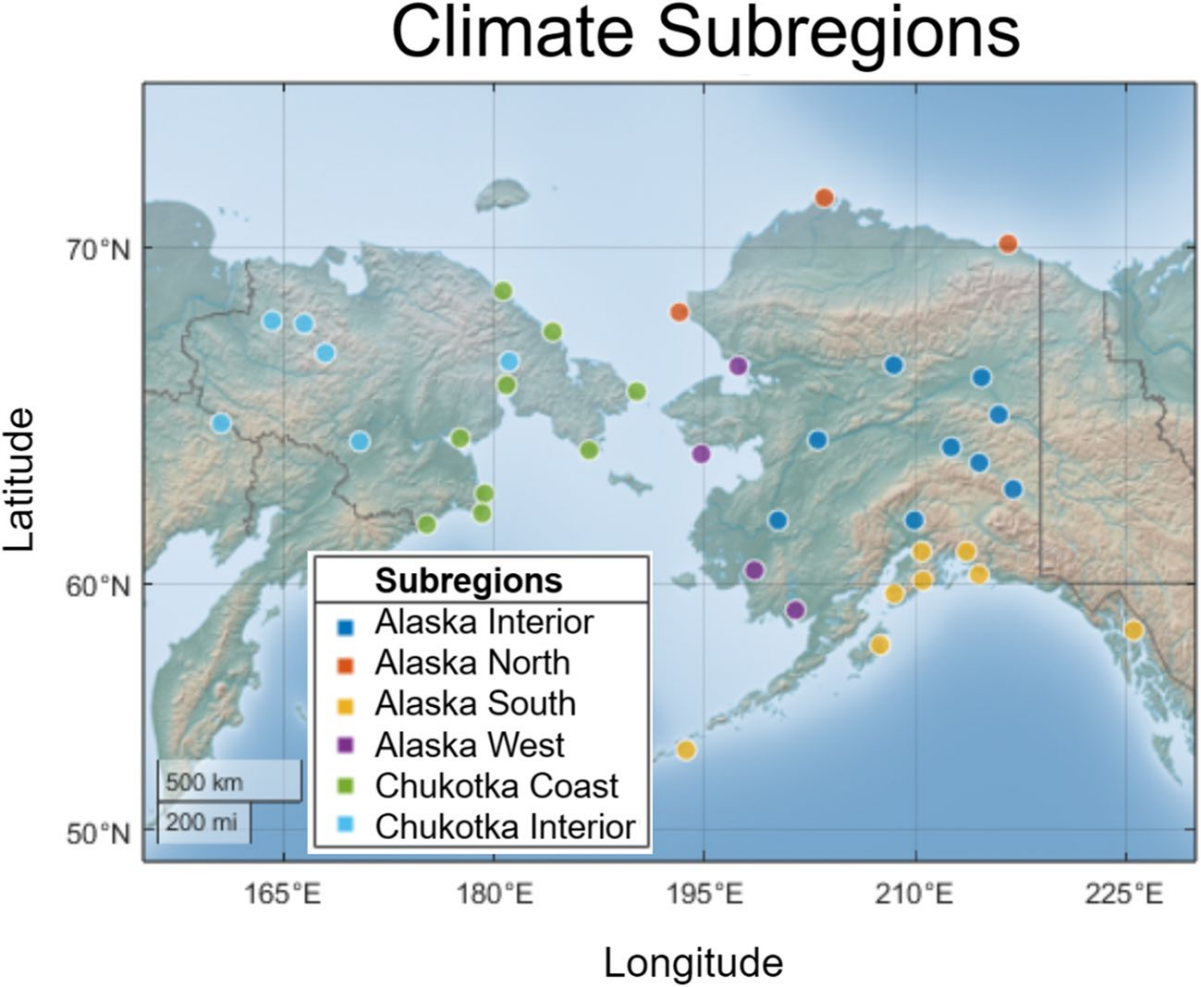
Study areas in Alaska and Chukotka. Selected locations (with weather stations) are marked with colored dots. The six subregions for which results presented are Alaska North (orange), Alaska West (purple), Alaska South (yellow), Alaska Interior (dark blue), Chukotka Interior (light blue), and Chukotka Coast (green)

The cold Arctic Ocean and the Bering Sea, and topography in Alaska and Chukotka, exert strong influences on the regional climates of Beringia (Fig. 1) (Bieniek et al. 2012; Akimov et al. 2014; Alexeev 2014; Mölders 2019; Walsh 2021). The Bering Sea branch of the warm Kuroshio Current flows along the coast of Alaska to the north, warming the western coasts of Alaska. Further north, it partially leaves through the Bering Strait, while another branch moves along the Asian coast to the south forming the cold Kamchatka current, cooling the eastern coasts of Chukotka (Akimov et al. 2014). Chukotka is mostly occupied by moderate (up to 1000 m high) Kolyma-Chukchi and Anadyr-Koryak mountain regions, with the highest mountain Velikaya (1888 m); lowlands are rare and, as a rule, are located near large lagoons in coastal areas (Fig. 1). In Alaska, the Pacific coast is separated from the interior by the Alaskan Range, with Denali (6190 m) as the highest point of North America. The interior consists of a plateau with a height of 1200 m in the east but generally below 600 m in the west, turning into lowland near the coast. The Brooks Ridge in the north has an east-west orientation, protecting the interior from the cold Arctic; it is followed by the Arctic lowland of the North Slope, which is opened to the influence of the cold Arctic Ocean with low temperatures and strong winds year-round (Fig. 1). As a result, Alaska’s more diverse relief and differences in the influence of the cold water bodies on both regions of Beringia are mirrored in a variety of climates (Bieniek et al. 2012; Mölders 2019; Walsh 2021). According to the Köppen–Geiger classification, the climate of Alaska changes from the Arctic Polar tundra in the north to subarctic oceanic and continental in the north-west and interior, and to temporal oceanic in the south and southeast. In Chukotka, the climate classification varies from Arctic Polar tundra in the north, to subarctic oceanic and continental in the north-east and interior (Peel et al. 2007; Beck et al. 2018).

The study area in the Arctic includes 39 locations in Beringia: 24 in Alaska, USA, and 15 in Chukotsky Autonomous Okrug (Chukotka), Russia. The locations were selected based on population size—as the most densely populated locations, touristic importance—as the locations most promising for the development of tourism (Drage et al. 2022; Schwoerer and Dawson 2022; Wienrich and Lukyanova 2022), and representativeness of the Köppen–Geiger subclasses. As the next step, they are grouped into smaller subregions. For both Alaska and Chukotka, interior and coastal subregions are identified in accordance with their climatological temperatures, which in turn are shaped by geography and topography. In particular, the major mountain ranges that separate the coastal areas from interior areas, such as Brooks Range, Alaskan Range in Alaska, and Anyu, Chukchi, Anadyr, and Koryak highlands in Chukotka, result in large temperature differences, especially in the seasonal range (Bieniek et al. 2012; Akimov et al. 2014). As a result, 39 locations were grouped into smaller subregions: North, West, South, and Interior in Alaska; Coast and Interior in Chukotka (Fig. 1, Table 1); Utqiagvik, Juneau, Anchorage, Fairbanks, Mys Shmidta, and Omolon are taken as the most populated and (or) the most indicative locations for each subregion, subsequently. For all 39 locations, the ERA5-HEAT pixel nearest to the geographical center of the location was selected for the analysis.

**Table 1:** Locations in Alaska and Chukotka and geographical coordinates

Study area	Subregion	Location	Latitude, N	Longitude, W(E)
Alaska	North	Utqiagvik	71°17′	156°47′W
		Kaktovik	70°08′	143°37′W
		Point Hope	68°21′	166°46′W
	Interior	Bettles	66°54′	151°31′W
		Fort Yukon	66°34′	145°15′W
		Circle	65°49′	144°04′W
		Galena	64°44′	156°54′W
		Fairbanks	64°51′	147°43′W
		Delta	64°03′	145°43′W
		Tok	63°19′	143°01′W
		Talkeetna	62°19′	150°05′W
		Holy Cross	62°12′	159°46′W
	West	Kotzebue	66°54′	162°36′W
		Nome	64°30′	165°24′W
		Bethel	60°48′	161°45′W
		Dillingham	59°03′	158°31′W
	South	Anchorage	61°13′	149°54′W
		Valdez	61°08′	146°21′W
		Cordova	60°33′	145°45′W
		Seward	60°07′	149°26′W
		Homer	59°39′	151°31′W
		Juneau	58°30′	134°42′W
		Kodiak	57°48′	152°24′W
		Unalaska	53°53′	166°32′W
Chukotka	Interior	Ostrovnoe	68°07′	164°10′E
		Bilibino	68°03′	166°27′E
		Ilirney	67°15′	167°58′E
		Amguema	67°01′	177°43′W
		Omolon	65°14′	160°32′E
		Markovo	64°41′	170°25′E
	Coast	Mys Shmidta	68°54′	179°22′W
		Mys Vankarem	67°50′	175°50′W
		Egvekinot	66°21′	179°07′W
		Uelen	66°10′	169°50′W
		Anadyr	64°47′	177°34′E
		Provideniya Bay	64°25′	173°14′W
		Beringovsky	63°03′	179°19′E
		Gavriila Bay	62°25′	179°08′E
		Khatyrka	62°03′	175°12′E

### Data

UTCI values for the period 1979–2020 are obtained from ERA5-HEAT reanalysis provided by the Copernicus Climate Change Service (C3S (Copernicus Climate Change Service) 2020; Di Napoli et al. 2020b; Hersbach et al. 2020). The procedure used in calculating the UTCI for ERA5-HEAT in C3S is based on meteorological variables (air temperature, dew point temperature, wind speed, air pressure, and radiation) and is summarized as follows. As a first step, the solar and thermal radiation fluxes at the surface of the Earth were extracted from ERA5 and used to calculate the mean radiant temperature (MRT) (Di Napoli et al. 2020a, 2020b, 2020c). Second, MRT, 2 m air temperature and relative humidity and 10 m wind speed were used as input into a multivariable equation to compute UTCI. The output is global (except for Antarctica) on a 0.25° spatial grid with 1 h temporal resolution over the period 1979–2020.

ERA5-HEAT is based on the ERA5 reanalysis. Because ERA5 is a model output constrained by data assimilation, there will be some discrepancies with observational data (e.g., synoptic station reports). However, there is a growing body of studies documenting the performance of ERA5 in high latitudes, and these studies generally cast ERA5 in a favorable light. In particular, ERA5 temperatures have been shown to compare well against observations over Arctic land and marine areas with reduced air temperature biases relative to other modern atmospheric reanalyses (Graham et al. 2019; Avila-Diaz et al. 2021). ERA5 has also been shown to be effective in capturing boreal high-latitude variation and trends of 2-m air temperature and precipitation (Barrett et al. 2020; Räisänen 2021). More specific to the Chukotka analysis in the present paper, recent studies have shown that ERA5 is also robust against observations and therefore represents a valid gridded product for the assessment of near-surface wind speeds over Alaska (Redilla et al. 2019). ERA5’s hourly analysis fields also provide an improved depiction of regional climate variables over alternative products such as those derived from the 5 km NOAA climate gridded dataset (Vose et al. 2014), as shown by Ballinger et al. (2023).

### Methods

The UTCI is based on the well-evaluated and advanced multi-node Fiala model of the human heat balance (Fiala et al. 2012; Psikuta et al. 2012), which is applied to assess the physiological response of the human body in a wide range of outdoor climatic conditions (Błażejczyk et al. 2012; de Freitas and Grigorieva 2017). The main categories of thermal stress, along with the corresponding physiological response and recommended protection measures, are listed in Table 2, which synthesizes results from previous research (Błażejczyk et al. 2012; Jendritzky et al. 2012; Di Napoli et al. 2019; Antonescu et al. 2021). The number of hours in each month of each year in the period 1979–2020 in each of ten UTCI categories was generated for the 39 locations in Alaska and Chukotka, using MatLab.

**Table 2:** UTCI values, thermal stress categories, physiological responses and protection measures, adapted from (Blazejczyk et al. 2012; Bröde et al. 2012; Jendritzky et al. 2012; di Napoli et al. 2019; Antonescu et al. 2021)

	UTCI range (°C)	Thermal Stress Category	Physiological responses*	Protection measures
1	Below −40	Extreme cold stress	Tre time gradient < −0.3 K/h. 30 min Tskfc < 0°C (frostbite)	Stay at home If outdoor exposure is necessary, use heavy and wind protected clothing
2	−27 to −40	Very strong cold stress	120 min Tskfc < 0°C (frostbite). Steeper decrease in Tre. 30 min Tskfc < 7°C (numbness). Occurrence of shivering. Tre time gradient < −0.2 K/h. Averaged Tskfc < 0°C (frostbite). 120 min Tskfc < −5°C (high risk of frostbite)	Intensify activity and protect face Extremities against cooling Use warmer clothing Reduce outdoor exposure time
3	−13 to −27	Strong cold stress	Averaged Tskfc < 7°C (numbness). Tre time gradient < −0.1 K/h. Tre decreases from 30 to 120 min. Increase in core to skin temperature gradient	Intensify activity and protect face Extremities against cooling Use warmer clothing
4	0 to −13	moderate cold stress	DTS at 120 min < −2. Skin blood flow at 120 min lower than at 30 min (vasoconstriction). Averaged Tskfc < 15°C (pain). Decrease in Tskhn. Tre time gradient < 0 K/h. 30 min. Tskfc < 15°C (pain). Tmsk time gradient < −1 K/h (for reference)	Intensify activity and protect face Extremities against cooling
5	+9 to 0	Slight cold stress	DTS at 120 min < −1. Local minimum of Tskhn (use gloves)	Use gloves and hat
6	+9 to +26	No thermal stress	Averaged sweat rate > 100 g/h DTS at 120 min < 1. DTS between −0.5 and +0.5 (averaged value). Latent heat loss > 40W, averaged over time. Plateau in Tre time gradient	Physiological thermoregulation Sufficient to keep thermal comfort
7	+26 to +32	Moderate heat stress	Change of slopes in sweat rate, Tre, Tskm, Tskfc, Tskhn. Occurrence of sweating at 30 min. Steep increase in skin wettedness	Drinking >0.5 L hr^−1^
8	+32 to +38	Strong heat stress	DTS at 120 min >+2. Averaged sweat rate > 200 g/h. Increase in Tre at 120 min. Latent heat loss >40 W at 30 min. Instantaneous change in skin temperature > 0 K/min	Use shaded places Drinking >0.25 L r^−1^ Temporary reduce physical activity
9	+38 to +46	Very strong heat stress	Core to skin temperature gradient < 1K (at 30 min). Increase in Tre at 30 min	Temporary use of air condition Shaded places necessary Drinking >0.5 L hr^−1^ Reduce physical activity
10	Above +46	Extreme heat stress	Increase in Tre time gradient. Steep decrease in total net heat loss. Averaged sweat rate >650 g/h, steep increase	Temporary body cooling Drinking >0.5 L hr^−1^ No physical activity

*DTS dynamic thermal sensation; *Tre* rectal temperature (°C); *Tskm* mean skin temperature (°C); *Tskfc* face skin temperature (°C); *Tskhn* hand skin temperature (°C)

#### Trend analysis

Time series were constructed for the number of hours in each category of UTCI for the whole study period 1979–2020, and for four decades (1980–1989, 1990–1999, 2000–2009, 2010–2019) separately; data from 1979 and 2020 were not included in the decadal analysis in order to construct equal-length (decadal) intervals. All trends were aggregated annually, seasonally and monthly, for each location, and averaged for each subregion. To evaluate trends in these time series, *R*^2^ was obtained from Excel analysis. The Mann-Kendall test was used to check significance of correlations and trends with a threshold probability of 0.05 (Mann 1945; Kendall 1975).

## Results

### Spatial distribution of thermal stress in Alaska and Chukotka

**Fig. 2:**
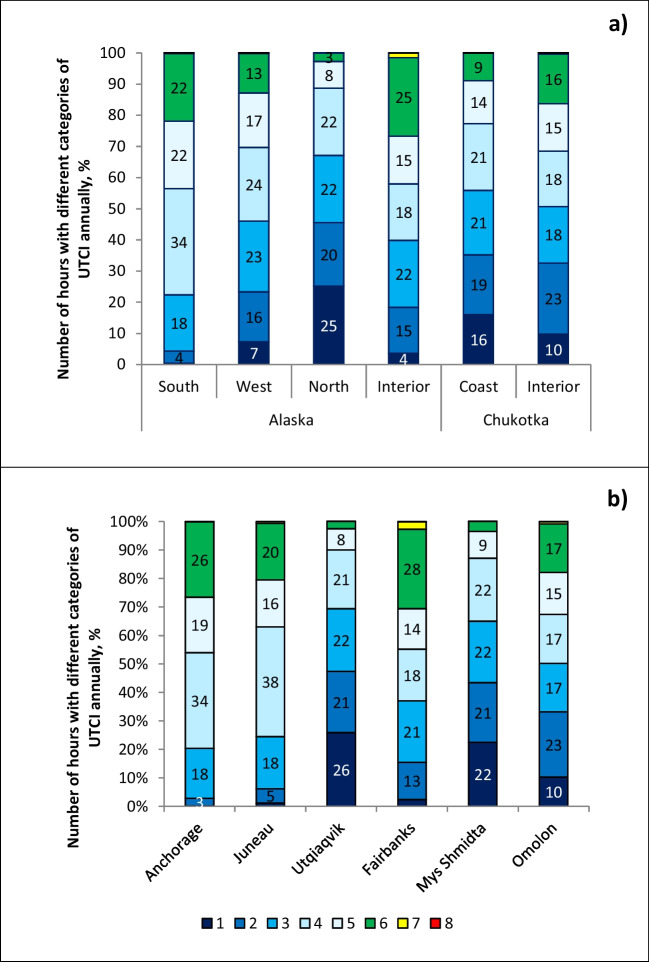
Percentage distribution of hours with UTCI in different categories of thermal stress over the full year from 1979 to 2020. Percentages are shown (**a**) as averages over all and (**b**) individual locations

Before addressing the seasonal variations and temporal trends of the UTCI, we first summarize the UTCI climatology by examining the annual distribution of the number of hours in each UTCI category. For each of the six climate regions, Fig. 2a shows this distribution, expressed as percentages of hours with UTCI in each category, averaged over the entire 1979–2020 period. Figure 2b shows the corresponding distributions for six specific locations—one in each subregion, in order to illustrate the climatologies at the community scale. While the full UTCI range contains ten categories, Fig. 2 demonstrates that the UTCI values in Alaska and Chukotka are essentially limited to eight categories, as UTCI categories 9–10 (UTCI >38°C) did not occur in these regions. Even category 7 (UTCI= 26°C…32°C) and more so category 8 (UTCI=32°C…38°C) occur with negligible frequency except in Alaska Interior, for which Fairbanks serves as a site-specific example in Fig. 2b. For the six subregions in Fig. 2a, the most common UTCI categories are category 4 (UTCI=–13°C…0°C), followed by categories 3 and 5.

The coldest category 1 (UTCI<−40°C) is most frequent in Alaska North, followed by coastal areas of Chukotka, with 25% and 16% of the hours, respectively. Utqiagvik in Alaska North and Mys Shmidta in Chukotka Coast, both of which are on the northern coasts of their respective landmasses, serve as examples, where 26% and 22% of hours, respectively, are in category 1. This category is not found in Alaska South, represented by Anchorage.

Northern subregions have the fewest hours in category 6: 2–3% in Utqiagvik and Mys Shmidta (Fig. 2b), and 3–9% in Alaska North and Chukotka Coast (Fig. 2a), respectively. At the same time, all other subregions and locations in Fig. 2 have at least 13% of their hours in the “no thermal stress” range. By the category 6 criterion, Alaska Interior is the most comfortable subregion with 25% hours of UTCI=9°…26°C (Fig. 2a). Alaska Interior’s representative community, Fairbanks at Fig. 2b, experiences 28% of UTCI in category 6. It is followed by the Alaska South (22%), represented by Anchorage (26%). It is interesting to note that Chukotka Interior (represented by Omolon) has fewer comfortable hours (16%) compared with interior parts of Alaska, which is discussed further in the “Discussion” section.

### Temporal changes of thermal stress in Alaska and Chukotka

**Fig. 3:**
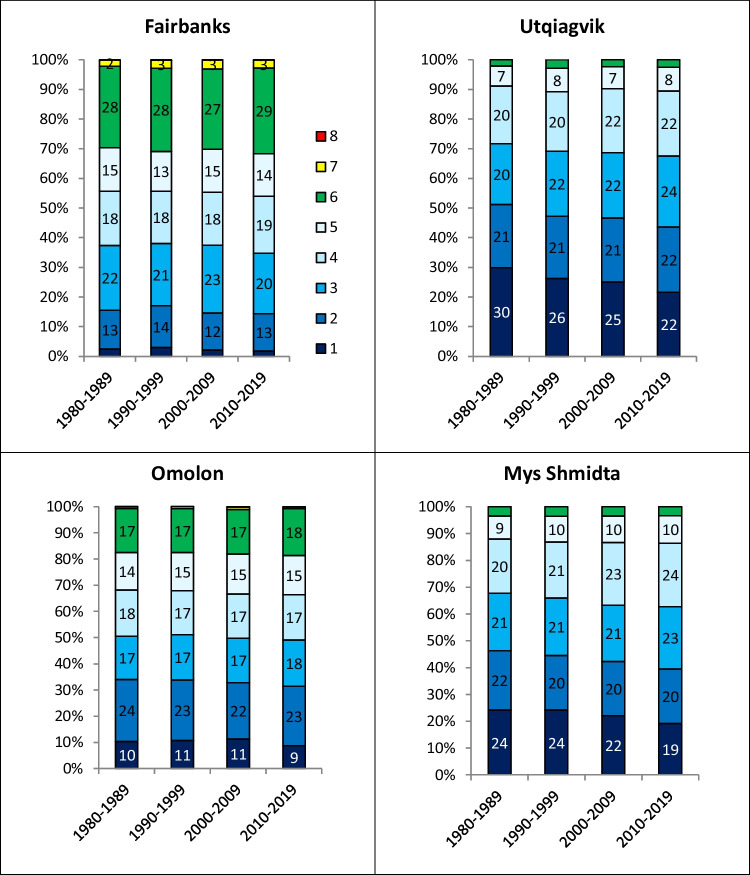
Percentage distribution of hours with UTCI in different categories of thermal stress, shown separately for four decades, %

A key objective of this study is the documentation of temporal changes in the UTCI distributions in Alaska and Chukotka. Figure 3 provides four examples of the evolution of the decadal climatology of the UTCI distribution from the 1980s to 2010s. Of these four locations, Utqiagvik (Alaska) and Mys Shmidta (Chukotka) are coastal locations with windier climates, while Fairbanks (Alaska) and Omolon (Chukotka) are inland locations at which high winds are less frequent, especially in winter ((Bieniek et al. 2012; Akimov et al. 2014; Alexeev 2014; Mölders 2019; Walsh 2021). Consistent with the climatologies in Fig. 2b, the higher frequencies of the coldest UTCIs (category 1) are apparent at the coastal locations. The two inland locations also have higher frequencies of the warmer categories 5 and 6. With regard to the temporal trends, the most salient feature of Fig. 3 is that all four sites show decreases of frequencies of category 1. When the final decade (the 2010s) is compared with the first decade (1980s), the category 1 frequency decreased by 3% and 4% at the coastal northern sites (Utqiagvik and Mys Shmidta), while the decreases were only about 1% at the inland sites. There were also increases in the frequencies of the milder categories at each location, especially in Utqiagvik and Mys Shmidta.

**Fig. 4:**
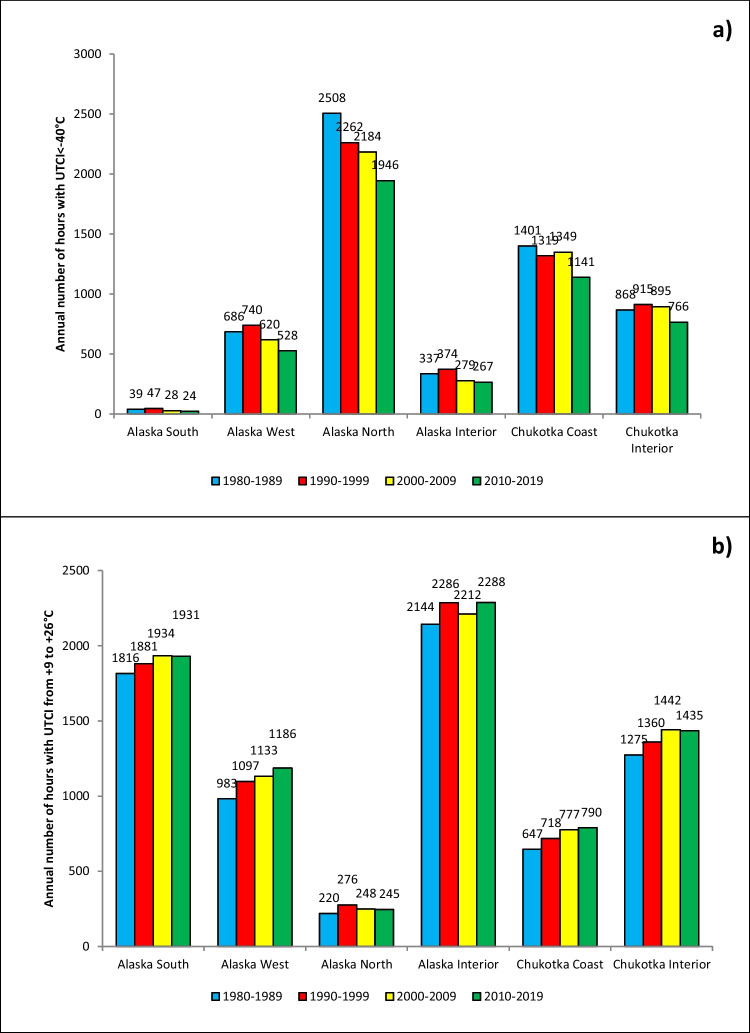
Average number of hours per year with UTCI in different categories of thermal stress: **a** < −40°C; **b** +9…+26°C

The trend toward less frequent occurrences of extremely cold (UTCI <−40°C) is even more apparent when the results are aggregated regionally, as shown in Fig. 4a. All regions show decreases of UTCI category 1 occurrences from the 1980s to the 2010s. As percentage changes, the decreases are especially large in the Alaska North (22.4%), Alaska West (23.0%), and Chukotka Coast (18.6%) climate subregions. In actual numbers of hours per year, the decrease in the Alaska North is the most pronounced, resulting in approximately 550 h/year. The corresponding value for the Chukotka Coast is a decrease of 260 h/decade. Even in the Alaska South, which rarely experiences UTCI <−40°C, the number of hours with the UTCI in this category decreased from 39 to 24 h per year from 1980–1989 to 2010–2019.

The decrease in the frequency of extreme cold has been accompanied by an increase in the number of hours with higher temperatures, including UTCI category 6 with “no thermal stress.” As shown in Fig. 4b, all regions have experienced an increase of category 6 occurrences, and this increase has been monotonic over the four decades in the Alaska West and Chukotka Coast. The increases from the 1980s to the 2010s vary greatly by subregion, ranging from 25 h/year in the Alaska North to 203 h/year in Alaska West. These gains of hours in the “comfortable” category have come largely from losses of hours in the coldest categories, as shown by the contrasting trends in the two panels of Fig. 4.

### Intra-annual variability and seasonality of UTCI in different categories of thermal stress

**Fig. 5:**
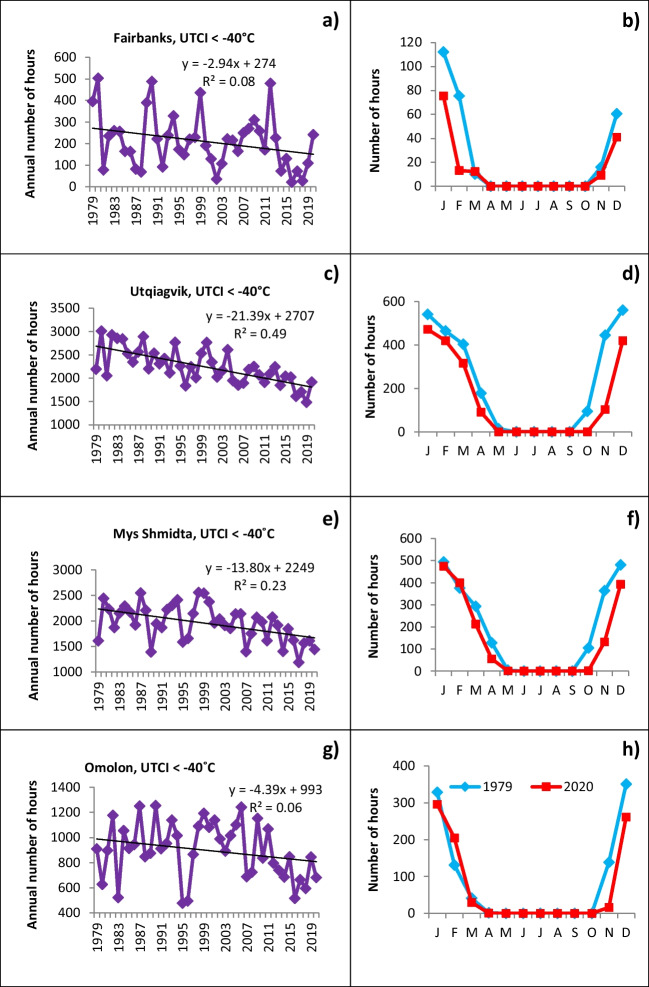
Number of hours with UTCI below −40°C (category 1): in Fairbanks (**a**, **b**), Utqiagvik (**c**, **d**), Mys Shmidta (**e**, **f**), Omolon (**g**, **h**). Left panels show interannual variations from 1979 to 2000; right panels show seasonal cycles for 1979 and 2020

**Fig. 6:**
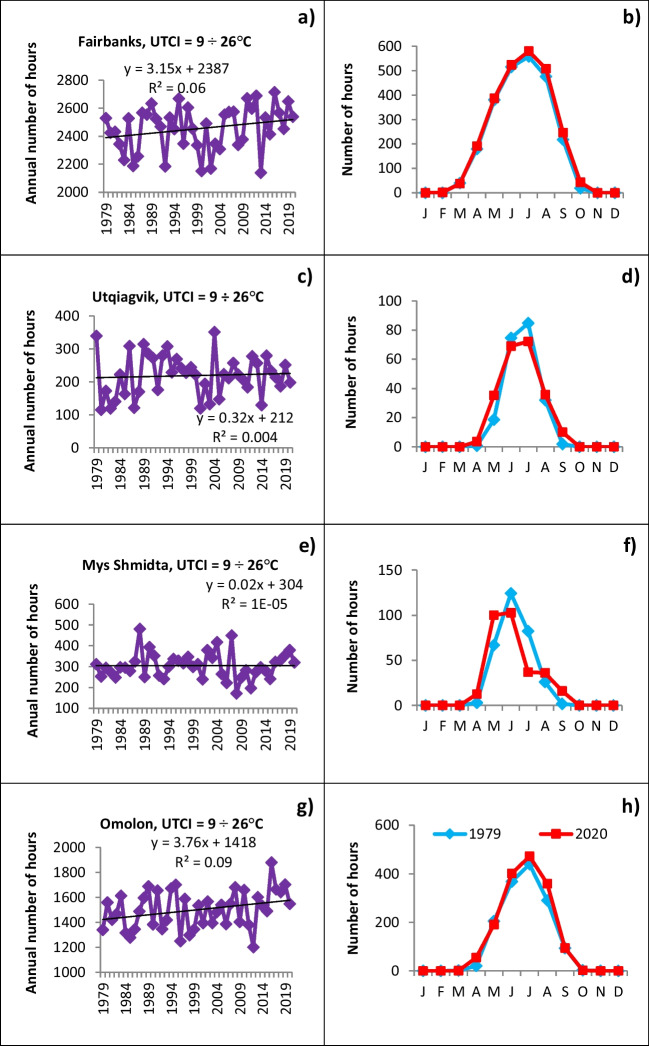
As in Fig. 5, but for UTCI category 6 (+9° to +26°C)

Figures 5 and 6 place the temporal trends into contexts of intra-annual variability and seasonality. In Fig. 5, each of the four sites discussed above shows year-to-year variations of several hundred hours in the coldest UCTI category. The interannual variations are especially large at the northern coastal sites. For example, Mys Shmidta has had yearly variability of UTCI totals in category 1 ranging from about 1200 to about 2550 h. Utqiagvik’s yearly total has ranged from less than 1500 h to more than 3000 h. Despite these large interannual excursions, the negative trends in the occurrences of category 1 of the UTCI are unmistakable at all the locations in Fig. 5.

The intra-annual distribution of UTCI in category 1 shows the obvious seasonality with its highest presence in winter months and an absence in summer. In the shoulder seasons, only March and November contain this category in all locations; April and October include category 1 in the coldest northern and coastal sites.

The seasonality of the interannual changes is highlighted in the right panels of Fig. 5, in which the blue and red lines show the UTCI values for the beginning and end points (1979 and 2020) of the trend lines of category 1 h in each calendar month. At the two northern coastal sites, Utqiagvik and Mys Shmidta, the largest changes have occurred in autumn and early winter. The changes in November are especially striking: Utqiagvik’s category 1 occurrences in November decreased from about 450 h in 1979 to fewer than 100 h in 2020. The corresponding values for Mys Shmidta are about 350 h in 1979 and 100 h in 2020.

Figure 6 demonstrates the inter-annual variations and seasonal changes of UTCI with the comfortable thermal perception, i.e., no thermal stress situations in the range from +9 to +26°C. A relatively high number of hours in this category are found in Interior subregions, both in Fairbanks (Fig. 6a), Alaska, and Omolon (Fig. 6g), Chukotka. The year-to-year variations are superimposed on growth through the whole study period. At the same time, Utqiagvik (Fig. 6c) in the Alaska North and Mys Shmidta (Fig. 6e) in Chukotka Coast have much fewer comfortable hours, with interannual variations comparable to their absolute values, and almost no temporal trend.

The seasonal distribution of comfortable temperatures shows an absence in winter months and late autumn, and peaks in summer. Spatial variations obviously appear in shoulder seasons: in the warmer interior, where the UTCI at Fairbanks can reach category 6 even in March and October (Fig. 6a), but only during April, May, and September at the coldest locations, Utqiagvik (Fig. 6d), Mys Shmidta (Fig. 6f), and even in Omolon (Fig. 6h) located in the Interior subregion of Chukotka. The right column of Fig. 6 shows the changes in the number of hours with UTCI in the zone with comfortable thermal perception from the beginning to the end points of the study period (1979 and 2020) at four locations. Slightly more comfortable hours in summer have been experienced in Interior subregions, both in Alaska and Chukotka, at the end of the study period compared to the beginning. Interestingly, the temporal increase in number of hours in the shoulder seasons is compensated by a decrease of comfortable UTCI in summer in the coldest locations, Utqiagvik and Mys Shmidta. As a result, there is almost no temporal trend overall in category 6, as noted above.

### Temporal dynamics of different categories of thermal stress: spatial distribution

**Fig. 7:**
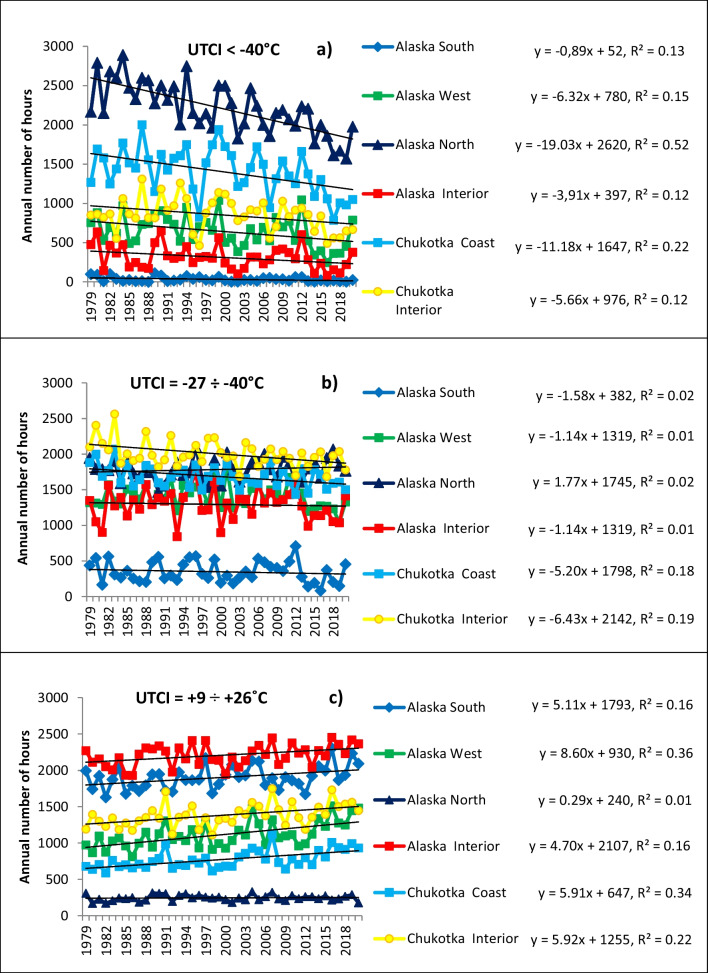
Temporal dynamic of number of hours with UTCI in different categories of thermal stress for subregions in Alaska and Chukotka: **a** UTCI<−40°C; **b** UTCI=−27…−40°C; **c** UTCI=+9…+26°C

Figure 7 and Fig. 1 (Suppl.) illustrate the temporal dynamics of the number of hours with UTCI in different categories of thermal stress for subregions in Alaska and Chukotka. Figure 1 (Suppl.) details the temporal changes through the whole study period 1979–2020 in all UTCI categories 1–8 within each region separately. For all subregions, the loss of hours with cold UTCI is compensated by an increase of hours with UTCI in warmer categories of thermal stress, and the temporal and spatial characteristics of their dynamics as summarized below.

In Alaska South (Fig. 1a, Suppl.), category 3 (UTCI=−27…−13°C) has the strongest negative trend with a decrease of about 3 h/year, which cumulatively leads to a reduction of up to 130 h over the entire study period. A very slow decrease can be found both for the coldest categories of UTCI (<−40°C, −40…−27°C), and −13…0°C. For UTCI >0°C, all categories 5–8 have positive trends. The largest increases, up to 214 h of increase over 42 years, are shown for the UTCI with the comfortable thermal perception. As a whole, a minor reduction of hours of UTCI with low temperatures is compensated by a slight growth of UTCI in categories 5–8, representing mild climatic conditions. Alaska West (Fig. 1b, Suppl) has the same trends, but with the highest negative trend found for the lowest (coldest) categories, which lose up to 6 h/year, and the highest positive trend of 8 h/year for hours with UTCI=+9…+26°C.

As mentioned above, the most noticeable changes compared to other subregions are depicted for Alaska North subregion (Fig. 1c, Suppl.) for the coldest UTCI <−40°C: its 800-h decrease was compensated by an increase in all other categories. Alaska Interior (Fig. 1d, Suppl) shows a loss of hours with UTCI<−13°C, with up to 4 h/year for UTCI <−40°C. This loss is compensated by a positive trend for all other categories.

Chukotka Coast and Interior (Fig. 1e and 1f (Suppl.), respectively) lose hours with UTCI=<−27°C; the largest decrease has occurred in Chukotka Coast, nearly 470 over the 42-year study period, which is very similar to Alaska North. This loss is compensated by positive trends in all other categories, with increases ranging up to 60 h/decade for UTCI with the comfortable thermal perception.

Figure 7 focuses the results on three key categories 1 (UTCI=<−40°C), 2 (UTCI=−40°…−27°C) and 6 (UTCI=+9°…+26°C). All subregions are shown on a single graph, separately for each category. For the coldest category 1, Fig. 7a shows the highest loss rate of hours for Alaska North, 19 h/year, followed by Chukotka Coast, 11 h/year; the changes are much less for all other subregions. The dynamics of hours with UTCI in category 2 is multidirectional (Fig. 7), with the fastest loss of hours in Chukotka, both Interior and Coast (6. and 5.2 h/year, respectively); there are negligible trends for Alaska South, West and Interior, and even a positive trend in Alaska North. All trends in category 6 are positive (Fig. 7), with the most noticeable growth for Alaska West, lower values in Chukotka, Alaska South and Interior, and a negligible trend in Alaska North.

Table 3 summarizes the temporal dynamics of the UTCI in different categories of thermal stress in all locations of Alaska and Chukotka for the entire study period from 1979 to 2020. These results are consistent with those described in Fig. 7 and Fig. 1 (Suppl), but with details for all locales separately. The significant negative trends (at 0.05 level according to Mann-Kendall test are colored in blue, and significant positive trends in red. The greatest loss of hours within the coldest range of UTCI has occurred at Utqiagvik (Alaska North) and Mys Vankarem (Chukotka Coast), −21.4 and −20.41 h/year, respectively. Almost no changes are shown for Unalaska (−0.08 h/year). The highest positive changes are shown for UTCI with the comfortable thermal perception for Nome in Alaska West (9.17 h/year) and Beringovsky in Chukotka Coast (8.93 h/year). The lowest positive change in this category has occurred at Utqiagvik, Alaska North (0.32). The most unpredicted result is the negative trend of the neutral category of thermal stress at Kaktovik (−2.90 h/year), which indicates a loss of the hours with comfortable UTCI in summer.

**Table 3:** Temporal trends (hours per year) of UTCI in different categories of thermal stress at locations in Alaska and Chukotka, 1979–2020. Trends in bold emphasis (negative) or italics (positive) are significant at 0.05 level according to a Mann-Kendall test (Mann 1945; Kendall 1975)

Study area	Location	<−40°C	−27 … −40°C	−13 … −27°C	0 … −13°C	+9 − 0°C	+9 … +26°C	+26 … +32°C	+32 … +38°C
Alaska									
North	Utqiagvik	**−21.40**	1.54	*8.99*	*7.72*	3.06	0.32	–	–
	Kaktovik	**−18.07**	2.69	*10.19*	*6.38*	1.94	**−2.90**	–	–
	Point Hope	**−17.58**	1.11	1.92	*4.63*	*6.68*	*3.46*	0.00	–
Interior	Bettles	−4.97	−1.05	−2.22	1.41	0.49	*6.07*	0.66	–
	Fort Yukon	**−6.96**	−3.35	2.64	2.49	0.93	3.44	1.46	0.09
	Circle	−3.53	2.37	−0.75	0.80	1.08	3.54	1.43	0.03
	Galena	−3.59	−3.91	−0.47	1.01	−0.69	*7.15*	0.07	0.17
	Fairbanks	−2.93	−1.07	−1.13	1.67	−0.36	3.15	1.09	0.09
	Delta	−2.11	2.65	−3.01	−1.16	−0.28	3.25	0.86	–
	Tok	−2.06	1.13	−2.33	−0.50	0.56	2.78	0.65	–
	Talkeetna	−1.64	−1.55	−4.21	1.46	−0.11	*4.75*	1.29	0.25
	Holy Cross	**−5.12**	−1.57	−0.65	−1.43	0.19	*8.21*	0.70	–
West	Kotzebue	**−10.40**	−3.26	0.96	**−4.24**	*8.57*	*8.86*	–	–
	Nome	**−6.45**	−3.43	−0.37	−0.79	2.06	*9.17*	0.10	–
	Bethel	**−6.33**	−0.63	−1.37	−1.85	2.10	*7.54*	0.00	–
	Dillingham	−1.98	−2.39	**−6.53**	1.68	−0.16	*8.82*	0.56	–
South	Anchorage	−0.20	−2.43	−4.05	−1.10	2.09	*5.59*	0.78	–
	Valdez	−1.26	−2.01	−2.59	−0.49	0.99	*5.24*	0.60	–
	Cordova	−1.87	−0.38	−2.39	−0.17	−1.19	*5.89*	0.85	–
	Seward	−0.72	−0.61	−4.68	−0.22	0.78	*5.18*	0.86	–
	Homer	−0.26	−2.35	−2.66	−2.76	1.51	*6.36*	0.72	–
	Juneau	−2.32	−2.13	−3.95	5.71	*2.06*	0.97	−0.06	–
	Kodiak	−0.34	−0.31	−1.90	−6.30	1.55	*6.91*	0.81	–
	Unalaska	−0.08	−2.13	−2.85	−2.34	2.09	4.74	–	–
Chukotka									
Interior	Ostrovnoe	**−7.04**	−5.42	1.07	1.08	*3.18*	*6.14*	*1.20*	0.01
	Bilibino	**−7.49**	**−7.05**	2.96	2.00	3.56	*5.89*	0.42	–
	Ilirney	−4.95	−5.30	0.84	0.88	1.71	*6.31*	0.56	–
	Amguema	**−9.58**	**−8.44**	0.85	*7.46*	3.95	*5.66*	0.43	–
	Omolon	−4.39	−3.06	1.02	−0.14	2.21	3.76	0.82	0.06
	Markovo	−3.83	**−6.64**	0.60	1.36	0.93	*6.18*	*1.32*	0.14
Coast	Mys Shmidta	**−13.80**	**−6.32**	4.42	*11.30*	*4.66*	0.02	–	–
	Mys Vankarem	**−20.41**	−0.33	*5.34*	*6.86*	*6.11*	*2.58*	0.02	–
	Egvekinot	**−12.67**	−5.34	1.07	*7.86*	1.73	*7.22*	0.34	–
	Uelen	**−15.61**	−2.76	*7.79*	*6.04*	*3.95*	0.66	–	–
	Anadyr	**−12.25**	**−6.26**	*5.28*	0.24	*3.77*	*8.74*	0.58	–
	Provideniya Bay	−4.56	**−9.63**	−0.64	3.34	*2.93*	*8.11*	*0.28*	–
	Beringovsky	**−9.94**	−5.47	1.93	−1.02	*5.63*	*8.93*	–	–
	Khatyrka	−4.57	−5.61	−0.46	1.85	−0.37	*8.77*	*0.51*	–
	Gavriila Bay	**−6.21**	−5.32	−2.18	2.62	2.78	*8.14*	*0.29*	–

**Fig. 8:**
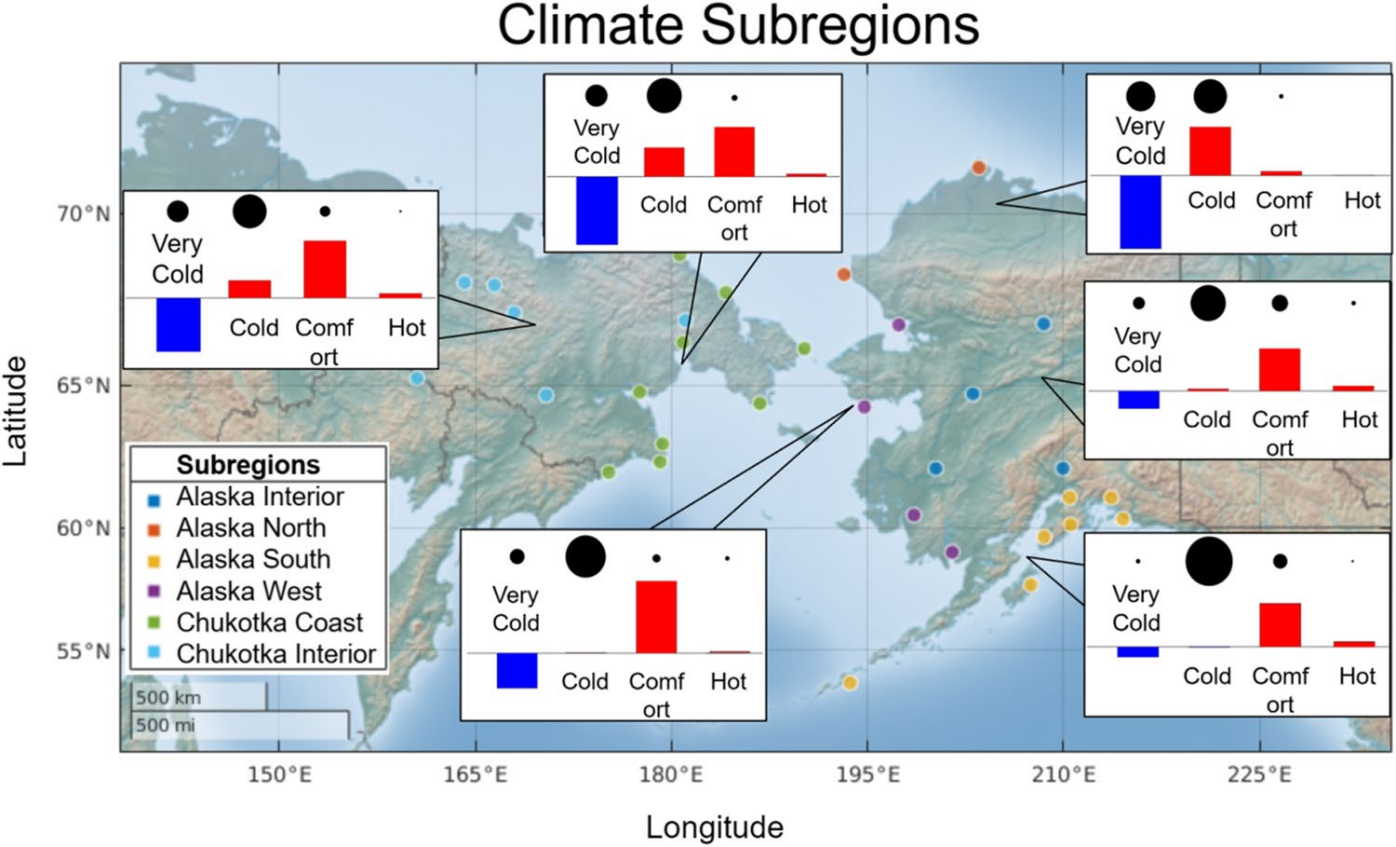
Summary of UTCI climatology and changes by subregions and consolidated categories. Thermal perception: “very cold,” categories 1+2; “cold,” categories 3+4+5; “comfortable,” category 6, “hot,” categories 7+8. Black circles’ diameters are proportional to climatological contribution percentages of the number of hours per year for each consolidated category. Trend bars are scaled according to values from Table 3 colored in blue for negative, red for positive trends

Figure 8 provides UTCI climatology and changes (trends) by subregions and simplified (consolidated) categories of thermal stress. For this summary, category groupings are as follows: “very cold” includes categories 1 and 2; “cold” includes categories 3, 4, and 5; “comfortable” includes category 6 only; and “hot” includes categories 7 and 8. Blue bars at Fig. 8 indicate negative trends for “very cold” situations everywhere in the study area, with the strongest decrease in Alaska North, followed by Chukotka Coast, Chukotka Interior, and Alaska West, Interior, South. The reduction of very cold temperatures is compensated by a positive trend in the “cold” categories for Alaska North; in both “cold” and “comfortable” for Chukotka Coast and Chukotka Interior; and in “comfortable” for all other regions. The highest positive shift of the “comfortable” perception has occurred in Alaska West, which is supported by the detailed results above. The results for all subregions show that the trend in “hot” perception is generally negligibly small but still positive.

## Discussions

The thermal state of the climatic environment can be expressed in terms of special indices that capture the impacts of environmental variations on humans. One such index is the universal thermal climate index (UTCI), a widely used bioclimatic index that combines effects of air temperature, humidity, wind speed and radiation, and both short- and long-wave, in a single value. This paper represents a detailed analysis of the UTCI in a framework of thermal stress categories, with a focus on temporal and seasonal dynamics over the Beringia region, which includes Alaska and Chukotka. The study period 1979–2020 is short enough to be comparable with the periods used to determine climate “normals,” and yet long enough for the assessment of recent variations and trends.

The first and the most noticeable feature of the dynamics in categories of thermal stress is the replacement of cold stress by those categories which are warmer, and most importantly, within the comfortable thermal perception zone, in almost all locations in the study area. This transfer points to a potential benefit of climate change for Beringia, one of the coldest regions on Earth, for people that live there and for the development of tourism in this area. The opposing trends represent one of the positive aspects of climate change for humans, although we underline that the findings pertain to regions with cold climates, in particular the Arctic. Warmer regions of the earth can be expected to experience a loss of “comfortable” hours as these hours shift to the categories of heat stress that are almost absent from the distributions for the regions of the present study. For example, Antonescu et al. (2021) found a significant increase in the annual number of hours with heat stress (UTCI > 32°C) in central and southern Europe in the period from 1979 to 2019 mainly due to the less frequent conditions with no thermal stress (Antonescu et al. 2021).

Some results obtained are especially remarkable. The first is the strong seasonality of cold stress, including its spatial extent, and its temporal variations. Another notable finding is the recent change in the occurrences of the more comfortable categories, for which both the spatial dimension and the seasonal duration have changed during the study period. Spatial differences between Alaska and Chukotka in both thermal stress distribution and its temporal trends merit further discussion, which can be found in the next paragraphs.

### Cold stress in Alaska and Chukotka

For the health of those people who live in the Beringia region and need to work outside for long periods, the most impactful thermal stress arises in UTCI<−27°C. These categories form the largest part of UTCI in the coldest subregions—in Alaska North and Interior Alaska and Chukotka, but negligible portions of the distribution in Alaska South (Figs. 2 and 3). Our findings are consistent with results from those researchers, who explored cold stress climatology for Alaska (Mölders 2019) and the northern areas of the Russian Far East (Vinogradova 2021) and the Russian north-west (Shartova et al. 2019). According to findings by Mölders (2019) for the study period from 1979 to 2017, both strong hear and extreme cold stress occurred recurrently in Interior Alaska. At the same time, the cold stress of various degrees was shown for the areas along the Arctic Ocean (Mölders 2019). Bioclimatic conditions in Arkhangelsk, located in the Russian Arctic, were explored by Shartova et al. (2019). Data inputs for years from 1999 to 2016 revealed comfortable period with no temperature stress for the months from June till September. Since Arkhangelsk is located in the area of Arctic with a strong influence of the warming Atlantic, there is no period with UTCI in categories of very strong and extreme cold stress; the coldest months of January and February experienced UTCI within the range of −13…−27°С, or a strong cold stress (Shartova et al. 2019).

Our results on temporal trends (Figs. 4–8, Fig. 1 Suppl) demonstrate a dramatic reduction of hours in the coldest range of UTCI, up to 25% in Alaska North, providing a prime example for discussion of those places in Beringia which can benefit from global warming. However, this region is so cold, with very low temperatures combined with strong wind that despite warming and reduction of values in categories 1–2, there are still a huge number of hours in the range of cold stress, which should be taken into consideration by local authorities in programs of social development.

Table 2 provides detailed information about the physiological responses of the human body in a cold thermal environment, as well as those protection measures which must be taken by a person (or advised by authorities for people) who live and work in cold regions, to escape negative consequences of cold stress on humans. To avoid a high risk of frostbite and (or) fall of skin temperature (face and hands), the important protective measures to be taken are just to escape an exposure, use special warmer clothing, or stay at home. The native peoples and those who have lived here for generations have already follow those protection measures, and are better adapted to cold environments both physiologically and in their behavior. Newcomers must take advantage of their experience.

### Seasonality of interannual changes

The dramatic temporal changes in hours with UTCI in the lowest category 1 in autumn are shown in the right panels of Fig. 5. These changes align closely with the reduction of autumn sea ice along the northern coasts of Alaska and Chukotka (Meier et al. 2021; Whaley et al. 2022). Whereas the sea ice edge was found at or near the coasts in most years through the 1980s and even into the 1990s, the past decade has been characterized by extensive areas of open water in the Chukchi and adjacent seas through much of autumn (Rolph et al. 2019). The enhanced absorption of solar radiation in summer is released to the atmosphere during the autumn freeze-up period, resulting in a moderating influence on air temperatures (Smith and Jahn 2019; Thomson et al. 2022). We can speculate, that as a result, UTCI<−40°C is becoming increasingly rare in the autumn months along the northern coasts. Because a sea ice cover is reestablished by January, and persists through spring, the changes in the frequency of extreme cold are much smaller at the coastal sites in the January–May period (Smith and Jahn 2019). By contrast, the months of the greatest reduction of UTCI in category 1 occurrences at Fairbanks, an inland site, are February, January, and December. In Chukotka, Omolon’s largest decreases have occurred in November and December, which was likely influenced by the diminished sea ice cover but here the effect was much less pronounced than in coastal locations. Further research is needed to test the relationship between sea ice cover seasonality and UTCI inter-year variability.

### Thermal stress and trends: Alaska versus Chukotka

From the climatological point of view, Alaska and Chukotka, as regions of Beringia, are very similar, but have some peculiarities, which are pronounced in both the climatology of thermal stress expressed by the UTCI, and by its temporal variations through the past four decades. Both regions extend north of the Arctic Circle and their coasts are washed by two main Arctic water bodies—the Arctic Ocean and Bering Sea. However, they are located at the opposite tails of the ocean currents, causing the main differences for the climate of the adjacent areas. Additionally, their mountain ranges differ in height and directions, affecting the climates of the inland locations differently in both Alaska and Chukotka.

Alaska North is the coldest subregion, open to the influence of the cold Arctic Ocean. Due to both very low temperatures and strong coastal winds, it experiences a quarter of all hours in the very cold category, and only 3% of its hours are in “no thermal stress” Category 6 (Fig. 2). Both subregions of Chukotka have lower frequencies of very cold hours: 16% and 10% in Chukotka Coast and Interior, respectively. It is interesting to note that Chukotka Interior has fewer comfortable hours compared with interior parts of Alaska, where a few hours (1%) are found in the hotter stress category 7. A possible reason may be the more pronounced influence of the cold Arctic Ocean on Chukotka, while the inner part of Alaska is isolated topographically by the Brooks Range to the north and the Alaska Range to the south.

Climate change manifests itself differently in various regions in the Arctic, depending on the main driving forces. Figure 8 shows that in Chukotka reduction of hours in very cold categories 1–2 is compensated by increase in both “cold” and “comfortable” thermal perceptions. In Alaska, these changes are more diverse both spatially and thermally. As the coldest subregion, Alaska North demonstrates a “warming” shift from “very cold” to “cold”, but to a “comfortable” range in Alaska’s Interior, West and South. Climate change in the northern regions, both in the northern part of the Coast Chukotka and in Alaska North, is largely caused by a decrease in the amount of multiyear sea ice in the Beaufort and Chukchi Seas, which reached its lowest level in the early 2010s and its second lowest level in the early 2020s (Meier et al. 2021). Additional research is planned to find the differences in spatial variations of not only air temperature, but humidity and wind, as the main components of thermal stress incorporated into the UTCI.

### Thermal comfort in Alaska and Chukotka: advantages for tourism development

Both Alaska Interior and Chukotka Interior and Alaska South experience comfortable temperatures during the period from April to October, and the occurrence of UTCI category 6 in Alaska Interior is as much as a quarter of all hours annually: our results show the 5–8 h increase annually, or up to 350 h during the entire study period, except in Alaska North. Our findings on the comfort range trends are compatible with the conclusions from Huang et al (2019) for the Arctic. They demonstrate an increase in UTCI from 1979 to 2019 at a rate of 0.457°C/10a, and spread of “comfortable” areas at a rate of 2.114*10^5^ km^2^/10a, and even with the higher rate of 6.353*10^5^ km^2^ over the last decade in the northern Brooks Mountains in Alaska (Huang et al. 2019).

In this respect, global warming brings the benefit of a lengthened comfortable interval in the Arctic. Assuming the comfort zone provides the best time for tourists to be outside, these outcomes have important implications for the development of many types of tourism, especially outdoor activities, such as skiing in spring and autumn, summer hiking, and sightseeing. But this trend can be offset and even interrupted by (i) wildfires, which can dramatically pollute air and diminish visibility, especially in Alaska Interior (Walsh et al. 2020); (ii) higher probability of heat waves in the Arctic as a whole (Walsh et al. 2020; Overland and Wang 2021), and in Beringia region, especially in Interiors of both Alaska and Chukotka; the latter is supported by our results showing an increase of UTCI frequency in categories 7 and 8; (iii) increased prevalence of blood-sucking insects and ticks: mosquitoes, black flies, and other outdoor pests (Cooke et al. 2002).

### UTCI and thermal comfort

Several further considerations are relevant to the concept of “comfort.” Thermal stress category 6 (“no thermal stress”) for 9–26°C, as used in this paper, shows a broad range of thermal “comfort” perception. However, it is not entirely consistent with the use of “comfort” range applied in other studies. In fact, it encompasses more than the Bröde et al.’s (2012) “thermal comfort zone” with UTCI ranging from 18 to 26°C. This narrower range is not considered separately in the current study, which can be focused in future. Another issue is the estimation of intra-day changes in UTCI. Since the UTCI gradation “no thermal stress” in high northern latitudes is most likely observed in summer during the afternoon or evening hours, it is worth knowing which categories prevail at night. Adding a diagnosis of the diurnal cycle for the warm season will be relevant, for example, for tourism and other outdoor activities during the Arctic summer.

## Conclusion

This study has provided a comprehensive evaluation of the spatiotemporal variability of a key climate-related metric of human comfort in the Beringia regions of the Arctic. The universal thermal climate index (UTCI) in northern latitudes has previously been evaluated in terms of its climatology and seasonality, but not in terms of trends based on a consistent data product such as ERA5-HEAT. The key findings of this study are the following:

-  The extreme coldest UTCI categories are most common in coastal locations of northern Alaska and Chukotka, where strong winds exacerbate the low temperatures during winter; the warmest UTCI categories are rarely reached in Alaska and Chukotka, and even category 7 is reached occasionally only at interior locations.
- The frequencies of occurrence of extreme cold have decreased up to 25% over the 1979–2020 period in Alaska and Chukotka.
- The number of hours in the comfortable UTCI category has increased substantially depending on subregion, from 25 to 203 h/year.

The increase in UTCI category 6 (comfortable thermal perception) occurrences and the decrease in the extreme cold UTCI categories (1 and 2) have positive implications for outdoor activities, from the perspectives of comfort and safety. However, offsetting factors, such as higher probability of wildfires and heat waves, and increased prevalence of blood-sucking insects and ticks, can accompany a warming climate.

Future work will include a diagnosis of the UTCI variations in terms of its component variables (temperature, wind, humidity, radiation), including an evaluation of the differences between the UTCI and the ambient air temperature. Another extension of the present study will be an evaluation of future changes in the UTCI as projected by climate models. In view of the changing historical distributions over the past several decades, substantial further changes can be expected in a warming world, depending on the rates of future changes in temperature, wind, humidity, and radiative fluxes, pointing to the need to utilize climate model output under alternative emission scenarios. Finally, an additional area for further study is the linkage between UTCI variations and socioeconomic impacts (health, economic, demographic). Such linkages may be detectable in the interannual variations as well as longer-term trends, although the availability corresponding socioeconomic data will likely limit the timeframe spanned by this type of interdisciplinary analysis.

### Supplementary information

ESM 1 (DOCX 64 kb)
