# Supplementary material for: Universal thermal climate index in the Arctic in an era of climate change: Alaska and Chukotka as a case study
Source: Int J Biometeorol. 2023 Aug 12;67(11):1703–21. doi: 10.1007/s00484-023-02531-2 (PMC10589199; doi:10.1007/s00484-023-02531-2)
Supplement: Supplementary file 1 — (DOCX 64 kb) [file 484_2023_2531_MOESM1_ESM.docx]

**Supplementary**

Universal Thermal Climate Index in the Arctic in an era of climate change: Alaska and Chukotka as a case study

International Journal of Biometeorology

Grigorieva E.A.^1*^, Alexeev V.A.^2^, Walsh J.E.^2^

^1^Humboldt Universitat zu Berlin

^2^International Arctic Research Center, University of Alaska Fairbanks

*corresponding author eagrigor3000@gmail.com

Grigorieva E.A. orcid.org/0000-0002-7811-7853, Humboldt Universitat zu Berlin (HU), Berlin, Germany, eagrigor3000@gmail.com

Alexeev V.A. orcid.org/000-0003-3519-2797, International Arctic Research Center, University of Alaska Fairbanks (IARC UAF), Fairbanks, USA, valexeev@alaska.edu

Walsh J.E. orcid.org/0000-0001-9541-5927, International Arctic Research Center, University of Alaska Fairbanks (IARC UAF), Fairbanks, USA, jewalsh@alaska.edu

|  |
| --- |
|  |
|  |
|  |
|  |
|  |

**Fig. 1** Trends of number of hours with different categories of UTCI, in Alaska: a) South, b) West, c) North, d) Interior; and Chukotka: e) Coast, f) Interior (criteria for Categories are given in Table 1, Supplementary)

|  |
| --- |
|  |
|  |

**Fig. 2** Temporal dynamic of number of hours with UTCI in different categories of thermal stress for subregions in Alaska and Chukotka: a) UTCI< –40°C; b) UTCI=–27÷–40°C; c) UTCI=+9÷+26°C
